# Cut off values for abdominal obesity as a criterion of metabolic syndrome in an ethnic Kyrgyz population (Central Asian region)

**DOI:** 10.1186/1475-2840-11-16

**Published:** 2012-02-22

**Authors:** Aibek E Mirrakhimov, Olga S Lunegova, Alina S Kerimkulova, Cholpon B Moldokeeva, Malik P Nabiev, Erkin M Mirrakhimov

**Affiliations:** 1 Kyrgyz State Medical Academy named by I.K. Akhunbaev, Akhunbaev street 92, Bishkek 720020, Kyrgyzstan; 2 National Centre of Cardiology and Internal medicine named by M. Mirrakhimov, T. Moldo 3, Bishkek 720040, Kyrgyzstan

**Keywords:** Obesity, Abdominal obesity, Waist circumference insulin resistance, Metabolic syndrome, Cardiovascular risk

## Abstract

**Background:**

People of different racial and ethnic backgrounds have a distinct pattern of central fat deposition, thus making it necessary to devise a race based approach for the diagnosis and evaluation of abdominal obesity (AO). This is the first study to determine the optimal waist circumference (WC) cutoff values for definition of AO in an ethnic Kyrgyz population.

**Methods:**

323 persons of Kyrgyz ethnicity (183 women and 140 men), with a mean age of 51.8 ± 9.5 years old were included in the study. Measurement of blood pressure (BP), anthropometric data (including body mass index calculation and WC measurement), fasting blood sugar, serum lipid parameters and insulin were performed in all examined individuals. Insulin resistance (IR) was considered as HOMA index (insulin × fasting glucose/22.5) ≥ 2.77. Sensitivity and specificity for the presence of IR or two other criteria of MS (according to the international classification, 2009) were calculated by using receiver operating characteristic (ROC) curves for men and women separately.

**Results:**

The optimal sensitivity and specificity obtained from the ROC curves for IR were 88 cm in women (sensitivity of 0.85, 95%CI (0.72-0.93), specificity of 0.58, 95%CI (0.49-0.66)) and 94 cm for men (sensitivity of 0.8, 95% CI (0.65-0.91), specificity of 0.61, 95% CI (0.51-0.71)). The data from the ROC curve for any two other MS criteria confirmed the results and the WC 88 cm in women (sensitivity of 0.82, 95% CI (0.72-0.9), specificity of 0.72, 95% CI (0.62-0.8)) and 94 cm in men (sensitivity of 0.74, 95% CI (0.62-0.84), specificity of 0.73, 95% CI (0.61-0.83)) were corresponded to the optimal sensitivity and specificity.

**Conclusion:**

WC ≥ 88 cm and ≥ 94 cm should be used as a criterion for the diagnosis of AO for Kyrgyz women and men respectively based on these results.

## Background

Metabolic syndrome (MS) is a particularly common medical condition consisting of abdominal obesity (AO), arterial hypertension, as well as disturbances in lipid and/or carbohydrate metabolism. The presence of MS increases cardiovascular morbidity and mortality and risk of future type 2 diabetes mellitus (DM) [1,2]. Obesity is believed to be a core pathologic stimulus for the development of hyperinsulinemia and insulin resistance (IR), which are predictive for the development of new onset type 2 DM [3]. Obesity is also associated with increased cardiovascular risk, but with age this association seems to fade [4].

Several clinical approaches for MS diagnosis are currently available such as, Adult Treatment Panel (ATP III) of the National Cholesterol Education Program [5] and its modified version [6] use waist circumference (WC) of ≥88 cm and ≥102 cm for the diagnosis of AO in women and men respectively. Since ATP III clinical criteria are best suitable for North American population, and the fact that people of Asian origin have higher abdominal fat with the same body mass index (BMI) as well as a greater risk for the new onset type 2 DM with the same BMI values and this approach may underestimate metabolic risk among Asians [7]. Dong and colleagues have found the greater accuracy of height to weight ratio for the diagnosis of MS compared to traditional measurement of WC in a Chinese population [8]. Interestingly, Park and colleagues have demonstrated that Asians with BMI < 27 kg/m2 have higher predictive power of epicardial fat for the presence of MS and coronary artery disease, which may indirectly support the notion of distinct pattern of adiposity among Asians [9].

The controversies of current clinical models for diagnosing MS were highlighted in the secondary analysis of the FIELD study [10]. The researchers compared three different criteria for MS: World Health Organization, ATP III and International Diabetes Federation. They have concluded that WHO MS criteria can identify patients with DM who have a lower risk of future cardiovascular events.

There are some misconceptions regarding the optimal WC values and methods of assessing this in some Asian populations [11,12]. A recent Joint Interim Statement on MS proposes that WC ≥80 cm and ≥90 cm are diagnostic for AO in Asian women and men respectively [13]. However such generalization may be wrong due to possible heterogeneities among Asian populations. Thus, the need for different diagnostic criteria based on ethnic background was critically needed.

The information about optimal WC values for MS diagnosis was not known for the Kyrgyz ethnic group, and this became a cardinal impetus for the study.

## Methods

### Studied population

323 individuals (183 females, 140 males) 30-80 years (mean age 51.8 ± 9.5 years) from the general population were included in the study. Informed consent was signed prior to enrollment. Exclusion criteria: pregnancy, concomitant thyroid disease, heart failure, chronic kidney and liver disease, insulin therapy and chronic alcohol addiction.

Systolic and diastolic blood pressure (SBP and DBP respectively) measurements, anthropometric assessment such as weight, height were measured in all study participants. WC was measured in the standing relaxed position, during expiration, at the midline between the lower costal margins and the iliac crest parallel to the floor. Body mass index (BMI) was calculated as a weight (kg) to height (m^2^) ratio.

The general characteristics of studied population are present in Table 1.

**Table 1:** General characteristics of studies population

Variables	Study participants (n - 323)
Age; years	51.8 ± 9.5
AH; n (%)	133 (41.2%)
SBP: mm Hg	135.2 ± 21.8
DBP; mm Hg	85.5 ± 12.5
Obesity (BMI ≥ 30 kg/m^2^); n (%)	97 (30%)
BMI; kg/m^2^	27.5 ± 4.8
WC; cm	91.7 ± 11.5
Smoking; n (%)	48 (14.9%)
Positive family history for CVD; n (%)	73 (22.6%)
Type 2 DM; n (%)	25 (7.7%)
Fasting glucose; mmol/l	5.92 ± 1.83
Insulin resistance; n (%)	88 (27.2%)
Serum insulin; μIU/ml*	7.25 (4.89-10.6)
HOMA index*	1.85 (1.18-2.99)
CHD; n (%)	28 (8.7%)
TC; mmol/l	5.12 ± 1.11
HDL-C; mmol/l	1.14 ± 0.34
LDL-C; mmol/l	3.27 ± 0.96
TG; mmol/l*	1.24 (0.95-1.92)

*Data is presented as median (25%-75%); Abbreviations: AH-arterial hypertension, BMI-body mass index, CHD-coronary heart disease, CVD - cardiovascular diseases, DBP-diastolic blood pressure, DM- diabetes mellitus, HDL-C-high density cholesterol, IR-insulin resistance, LDL-C-low density cholesterol, SBP-systolic blood pressure, TC-total cholesterol, TG-triglycerides, WC-waist circumference

### Laboratory tests

Blood collection from the cubital vein took place after 12 h of fasting in the morning, with further separation to the plasma and serum, which were frozen in liquid nitrogen. Subsequently laboratory tests such as fasting plasma glucose (FPG), total cholesterol (TC), triglycerides (TG), high density lipoprotein cholesterol (HDL-C) and immunoreactive insulin levels were analyzed from the frozen blood samples. Low density lipoprotein cholesterol (LDL- C) was calculated by Friedwald W. formula [14]. Homeostasis model of assessment (HOMA) was used as a surrogate marker of IR and calculated as serum insulin (μIU/ml) × plasma glucose (mmol/l)/22.5. HOMA index ≥2.77 was considered to be diagnostic for IR, according to the results from the Bruneck study [15].

### Statistical analysis

The "Microsoft-Statistica 8.0" and "Graph Pad PRIZM 5" software were used for statistical analyses. ANOVA according to Kruskal-Wallis method was used to compare the IR values (given the nonparametric distribution of variables). A posthoc comparison of variables was measured by the Mann-Whitney test with Bonferroni correction. Odds ratio (OR) and 95% confidence interval (CI) were calculated using the "2 × 2 tables" in the "Graph Pad PRIZM 5" program. Receiver-Operating Characteristic (ROC) analysis was performed with the construction of ROC curves to identify the borderline WC values. Variables are present as mean ± standard deviation for normal distribution and median (25%-75%) for variables with nonparametric distribution. The p value < 0.05 was used as a cut off for statistical significance.

## Results

To define the threshold values for WC, the entire data set (separately for men and women) was divided into sub-groups with WC increments of 4-5 cm. Common borderline values from the modified ATP III criteria (≥ 102 cm in men and ≥88 cm in women), the International diabetes Federation (IDF) criteria (≥94 cm in men and ≥80 cm in women) and the IDF criteria for the Asian population (≥ 90 cm in men and ≥80 cm in women) were taken into consideration. Thus, women were subdivided based on the WC values into subgroups of < 80 cm, 80-87 cm, and ≥88 cm; and men were subdivided into these subgroups < 90 cm, 90- 93 cm, 94-101 cm, ≥102 cm.

After this, each subgroup was analyzed for the presence of IR, blood insulin concentration, the HOMA index and the OR of having IR. A greater WC was associated with an increased likelihood for IR in both women and men. Women with WC ≥88 cm had 2.5 times increased prevalence of IR compared to the WC of 80-87 cm. 23% of men with WC ≥90 cm had IR, whereas 28% of men with WC of 94-101 cm had IR. 60% of men with WC ≥ 102 cm had IR, which was expected. However statistical significance was observed only for the subgroup with WC of 94-101 cm.

The gradual statistically significant increase of HOMA index and blood insulin concentration was found in association with the increase in WC for all groups. Neither men nor women experienced jumps in HOMA index or insulin concentration.

Greater OR for IR were associated with the increase in WC. Statistically significant differences in OR for IR were observed between the first and third women subgroups. Whereas differences between the first and second women subgroups were close to statistical significance. Men had statistically significant differences in OR for IR compared to men with WC < 90 cm. The OR for IR increased in men with WC of 94-101 cm, but compared to the WC of 90-93 cm the difference was not significant (Tables 2 and 3).

**Table 2:** IR indexes according to WC in women

Subgroups	WC; cm	IR; n (%)	**Insulin concentration**^**$**^	**HOMA**^**$**^	OR (95% CI)
1	< 80 (n-41)	1(2.4)	4.49 (2.83-5.92)	1.03 (0.63-1.43)	1

2	80-87 (n-40)	6(15)	6.74* (5.39-8.41)	1.64*(1.21-1.96)	7.05(0.8-61.6) P_1-2 _= 0.057

3	≥88 (n-102)	40(39)*^#^	9.04*^# ^(7.10-14.0)	2.46*^# ^(1.79-3.71)	26 (3.4-19.5) P_1-__3_ < 0.0001 P_2-3 _< 0.005

	P	< 0.00001	< 0.0001	< 0.0001	

$- Data is presented as median (25%-75%);

*p < 0.0001 as compared with 1 subgroup; #p < 0.0001 as compared with 2 subgroup Abbreviations: OR-odds ratio

**Table 3:** IR indexes according to WC in men

Subgroups	WC; cm	IR; n (%)	**Insulin concentration**^**$**^	**HOMA**^**$**^	OR (95% CI)
1	< 90 (n-44)	2 (4.2)	3.85 (2.69-6.87)	0.95 (0.65-1.65)	1

2	90-93 (n-21)	5 (23)	6.03 (5.14-7.05)	1.49 (1.30-2.08)	6.56 (1.15-3.73) P_1-2 _< 0.01

3	94-101 (n-35)	10 (28)*	7.41* (5.32-10.91)	1.87* (1.38-3.04)	8.4 (1.7-4.15) P_1-3 _< 0.003

4	≥102 (n-40)	24 (60) *^#£^	10.7*^#£ ^(8.3-15.65)	3.14*^#£ ^(2.17-4.81)	31.5 (6.66-14.9) P_1-4 _< 0.0001 P_2-4_001 P_3-4 _< 0.005

	P	< 0.00001	< 0.0001	< 0.0001	

$-Data is presented as median (25%-75%)

*P < 0.0001 as compared with 1 subgroup; #P < 0.0001 as compared with 2 subgroup; £P < 0.0001 as compared with 3 subgroup

**Figure 1:**
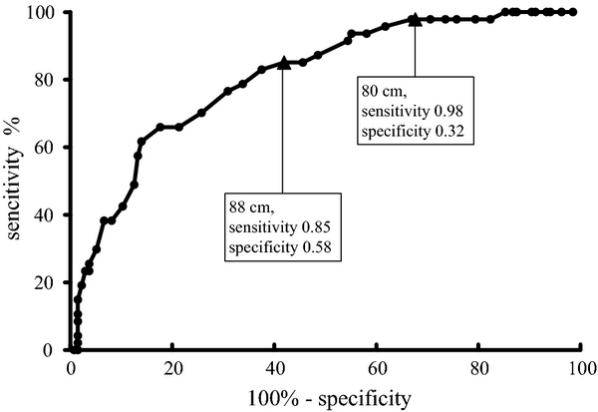
**Sensitivity and specificity for the presence of IR according to WC increase in women (ROC curve)**.

During the construction of ROC curves, women with WC of 80 cm had a sensitivity of 98%, but relatively low specificity of 32%. Whereas women with WC of 88 cm had specificity of 58% and sensitivity of 85% (Figure 1).

**Figure 2:**
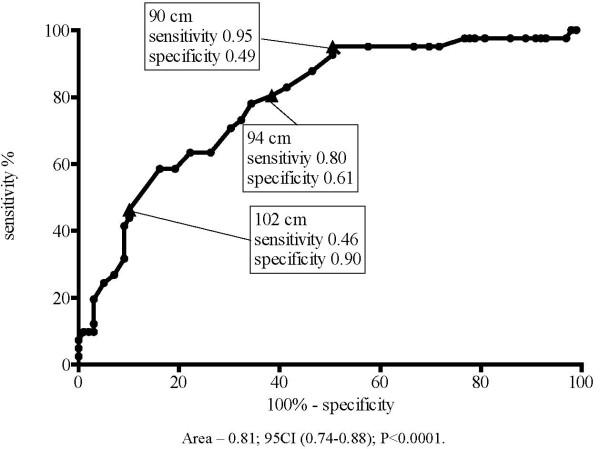
**Sensitivity and specificity for the presence of IR according to WC increase in men (ROC curve)**.

In an analogous analysis, men with WC of 90 cm had a sensitivity of 95% and relatively low specificity of 49%. Men with WC of 94 cm had greater specificity of 61% and sensitivity 80%, and men with WC of 102 cm, had specificity of 90%, but the sensitivity was markedly decreased and reached 46% (Figure 2).

Current clinical guidelines do not include IR as the MS criteria. Therefore, we also conducted ROC analysis to identify the threshold WC values, which increase the risk for detection of any two other MS criteria (BP ≥ 130/85 mm Hg, fasting blood sugar ≥ 5,6 mmol/l, HDL-C in men < 1.03 mmol/l and in women < 1.29 mmol/l, TG 1.7 mmol/l).

**Figure 3:**
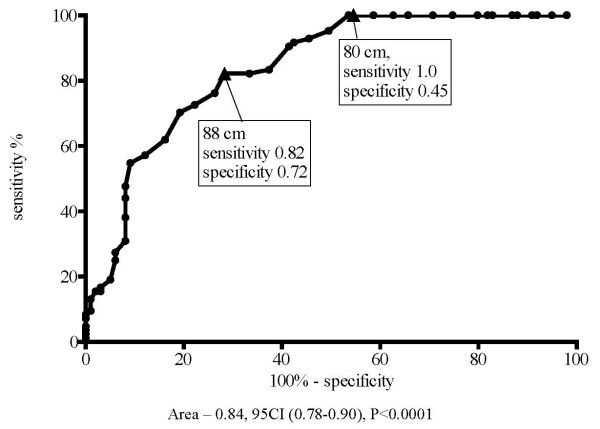
**Sensitivity and specificity for detection of two MS criteria in women (ROC curve)**.

The results of this analysis were almost the same as during the ROC analysis for the IR. Thus, for women with WC of 80 cm the sensitivity was nearly 100%, but specificity was only 45%. Women with WC of 88 cm had a moderate decrement in sensitivity to 82%, and increase in specificity to 72% (Figure 3).

**Figure 4:**
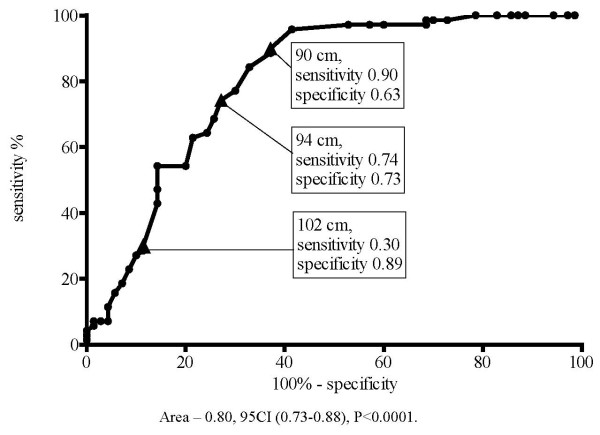
**Sensitivity and specificity for detection of two MS criteria in men (ROC curve)**.

In men with the WC of 90 cm a fairly high specificity (63%) was noted, which was increased to 73% in men with WC of 94 cm and men with WC of 102 cm had specificity of 89%. Men with WC of 94 cm had moderately decreased sensitivity of 74%, and men with WC of 102 cm experienced a sharp drop in sensitivity to 30% (Figure 4).

## Discussion

The data regarding the definition of AO based on ethnic and racial guidelines is controversial. US Caucasians have increased cardiovascular risk with WC ≥102 cm and ≥88 cm for men and women respectively. Whereat, African Americans have borderline WC of 89 to 109 cm for men and from 83 to 105 cm for women respectively, due to a lower amount of visceral fat with the same BMI [16-18]. Since African Americans have a higher prevalence of cardiovascular risk factors it was believed to use a similar WC cut off. The similar conclusions were made for indigenous American population [19].

The same is true regarding borderline WC values for Europeans. Accordingly to some studies, the risk of new onset type 2 DM development increases from WC ≥94 cm and ≥ 80 cm in men and women respectively [20-22].

The studies on Asian populations have shown that Asians have a higher level of visceral fat within the same BMI values compared to Caucasians [23,24].

In India, where type 2 DM and IR are highly prevalent [24,25], the researchers have found that the risk of type 2 DM started to rise from BMI > 23 kg/m2 and WC > 85 cm in men and WC > 80 in women [26,27]. The optimal WC values of ≥85 cm for men and ≥80 cm for women of Chinese descent were found after pooling the results of 13 population studies [28].

Similar studies were performed in Japan where borderline WC values of 85 to 90 cm for men and > 80 cm for women were found [29]. In an ethnic Korean population, the borderline WC ranges from 85 to 90 cm in men and from 80 to 85 cm in women [30-32]. The borderline WC values of 99.5 cm for men and 94.3 cm were found for Iranians aged 19-65 years [33].

Controversies on the optimal definition of the AO according to the racial and ethnic background are still present. Our data may be extrapolated on nearby Central Asian indigenous groups, such as Kazakhs, Uzbeks and Turkmens and help to resolve some of the current difficulties in this area. Nevertheless studies with a larger population sample are critically needed to find the optimal definition for AO in Central Asian population groups. This approach can significantly increase the accuracy for the diagnosis of AO, which is a key criterion for the diagnosis of MS. Thus it can potentially decrease the healthcare burden of this disease through appropriate lifestyle and medical intervention in a proper time.

## Conclusion

This is the first study to determine the optimal WC cut off values for an ethnic Kyrgyz population. WC ≥ 88 cm and ≥94 cm should be used as a criterion for the diagnosis of AO for Kyrgyz women and men respectively based on these results.

## Abbreviations

AO: Abdominal obesity; ATP: Adult treatment panel; BMI: Body mass index; DBP: Diastolic blood pressure; DM: Diabetes mellitus; FPG: Fasting plasma glucose; HDL-C: High density lipoprotein cholesterol; HOMA: Homeostasis model of assessment; IDF: International diabetes federation; IR: Insulin resistance; LDL-C: Low density lipoprotein cholesterol; MS: Metabolic syndrome; ROC: Receiver operating characteristic; SBP: Systolic blood pressure; TC: Total cholesterol; TG: Triglycerides; WC: Waist circumference.

## Competing interests

The authors declare that they have no competing interests.

## Authors' contributions

All authors contributed equally in the patient evaluation and article draft composition. AEM and OSL performed statistical analysis of the data. EMM edited the manuscript for content and AEM revised it and translated into English. All authors read and approved the final manuscript.
